# Dr. Henry Bennett on the Curability of Consumption, Etc.

**Published:** 1867-02

**Authors:** 


*Dr. Henry Bennett on the Curability of Consumption, etc.—Mr. Lockhart Clarke's Investigations of the State of the Spinal Cord in Tetanus, etc.*

LONDON, December, 1866.

A valuable contribution to medical literature, has lately been made by Dr. Henry Bennett, on the curability of consumption. His remarks are all the more interesting, because they come from one who, himself, is a sufferer from the disease. Seven years ago, Dr. Bennett was in extensive practice in London as a physician, and had made himself a name by his investigations and writings on obstetric medicine. His position here, however, he was compelled to throw up from ill health, and he went, as he says, a consumptive invalid to the South of France to die. But when released from the cares and labours of professional life, and placed under most favorable hygienic conditions in his new home in Mentone, he gradually improved in health, and now is able to give us the results of a large experience there acquired, in the treatment and prognosis of phthisis.

He seems to have regarded phthisis from the first, not as a disease per se, but, as he expresses it, merely a symptom grafted upon a general decay, just like a fungus on an unhealthy tree; and, leaving the disease out of consideration, he has made it his object to combat with the decay as the real source of the disease, just as the gardener might attend to manuring and strengthening the tree to destroy its parasites. The chief arguments which he adduces in favor of this doctrine, are:—the deleterious effects of privation, impure air and other deteriorating influences in producing and accelerating tubercular disease; and the beneficial results of treatment, when directed to sustaining the vital energies of the patient. He instances the natives of Labrador, who, in their rude, imperfect huts, exposed to many hardships, are almost unacquainted with the disease; but when they come down the St. Lawrence, and are crowded in the badly ventilated dwellings at the fisheries, yet living in comparative luxury, fall victims in a year or two to phthisis under these enervating influences.

Now, it would seem, if the view were correct that phthisis is merely a symptom of decay, that it ought to occur much more frequently in the aged. As a matter of fact, however, it is rare to find it in an incipient, or even active condition in them, but on the contrary, its active ravages are mainly confined to persons about the age of puberty and of middle life.

Recent investigations, too, seem to indicate, at least, the great probability that two or more distinct diseases, have been included under the common designation phthisis—that grey or miliary tubercle is distinct from common yellow or scrofulous tubercle, and that both of these are to be distinguished from certain forms of chronic inflammatory degeneration. Whatever may be said of the yellow tubercle, the first kind at least—the miliary tubercle or "gray granulation" appears to possess distinctive and specific characters, which give it the right to be considered as a separate disease; for experiments have been recently made, which appear conclusively to prove that tubercle of this kind, from a phthisical patient may by inoculation be made to communicate the disease to a healthy animal.

Mr. Villemin reports in the *Gazette Hebdomadaire*, that he has, again and again, succeeded in inoculating rabbits with tubercle, and that the tubercular matter thus produced in one rabbit, can be, in like manner, transmitted to another. Mr. Simon has lately made a similar series of experiments at St. Thomas's Hospital, with the following results:—Of thirteen rabbits inoculated with recent tubercle (taken from twelve to twenty hours after the death of the patient,) five were found to have deposits in the lungs, five months afterwards, and these deposits, when examined, were found to have all the appearances, general and microscopical, of tubercle. Another series has been inoculated from this tuberculous deposit, and I hope to be able to give you the result of this farther experiment at a future period. The rabbits were not subjected to any deteriorating influences.

While, therefore, we cannot allow the entire truth of Dr. Bennett's theory, that phthisis is a mere symptom, since we have good evidence that, at all events, in some cases, it is a specific disease; yet we may go with him to the extent of believing that depressing influences greatly favor the occurrence and progress of the disease, and as we know of no specific remedies for tubercle, our treatment must mainly be directed to combatting these influences, and thus will entirely coincide with his own. In fact, in phthisis, as in other diseases of an adynamic type, the main indication is to support the "vital energy" of the patient. To this end, we do not recommend the emigration of phthisical patients to relaxing or tropical climates, where the energies are exhausted, and where the mortality from this disease is proved to be larger than in an unequal climate like our own; but we rather send them to warm but bracing localities, of which Mentone, in the South of France, seems to be an admirable specimen. Though the place is full of consumptive invalids, the mortality from pulmonary phthisis, there stands at 1 in 55, while in London and Paris it is 1 in 8. The importance of hygiene in this disease, cannot be over estimated, and perhaps the most important thing of all, is good equable ventilation, which is, in our climate, difficult to obtain, and which deserves much more attention than it has hitherto received. In many cases, pure air is the food par excellence, upon which the patient can exist, and to compel or allow him to breathe a close atmosphere, loaded with effete organic impurities, when the quantity of active lung tissue is reduced to a minimum, is a scientific crime equal to manslaughter.

The value of different kinds of food, is an interesting subject, upon which I must not be led away here, farther than to give Dr. Bennett's opinion of fats. Cod-liver oil he looks on as undoubtedly only a readily digestible oil. Its strength-giving properties have been abundantly proved, while the number of cases of phthisis cured or relieved by treatment with this medicine, is far larger than of those treated by any other remedy. It is remarked also, in the *Intellectual Observer*, (for July of this year) that the Tyrolese chamois hunters prefer to take beef fat, rather than meat, with them, when exposed to lengthened fatigue in the mountains; and recent experiments tend to prove that "the production of muscular power is not so much to be attributed to the assimilation of nitrogenous food, as to the slow combustion of carbonaceous food." This is a very important matter, and deserving of farther investigations, since it bears not only upon phthisis, but also upon various other diseases accompanined by low vitality.

Dr. Bennett makes all his consumptive patients, if they are strong enough, use a sponge bath daily, at a temperature of about 65° F., and finds it of immense benefit. He says, "I myself, derived the greatest possible comfort and benefit from cold sponging in Summer, in the open air, on the banks of a Scotch loch, the waters of which were at 60°; and that when I was very ill, pulse 100 and skin hot and feverish. This gave me a confidence I have never lost, and which I have never had reason to repent."

I believe that he is undoubtedly right, when he remarks, that "much may be done towards arresting the progress of this decay, and even towards effecting a cure, by the combined influence of hygiene, of climate, and of rational medical treatment," and it is pleasing to find him able to add, "I am now surrounded by a little tribe of friends and patients, whose lives have been saved, like my own, by the steady application of these principles."

---

A Morgue in New Orleans.—They are to have a morgue in New Orleans, similar to that in Paris, New York, and other cities.

